# The Adverse Effects of Prenatal METH Exposure on the Offspring: A Review

**DOI:** 10.3389/fphar.2021.715176

**Published:** 2021-07-14

**Authors:** Jia-Hao Li, Jia-Li Liu, Kai-Kai Zhang, Li-Jian Chen, Jing-Tao Xu, Xiao-Li Xie

**Affiliations:** ^1^ Department of Forensic Pathology, School of Forensic Medicine, Southern Medical University, Guangzhou, China; ^2^ Department of Forensic Clinical Medicine, School of Forensic Medicine, Southern Medical University, Guangzhou, China; ^3^ Department of Toxicology, School of Public Health (Guangdong Provincial Key Laboratory of Tropical Disease Research), Southern Medical University, Guangzhou, China

**Keywords:** methamphetamine, psychostimulant, prenatal exposure, offspring, adverse effects

## Abstract

Abuse of methamphetamine (METH), an illicit psychostimulant, is a growing public health issue. METH abuse during pregnancy is on the rise due to its stimulant, anorectic, and hallucinogenic properties. METH can lead to multiple organ toxicity in adults, including neurotoxicity, cardiovascular toxicity, and hepatotoxicity. It can also cross the placental barrier and have long-lasting effects on the fetus. This review summarizes neurotoxicity, cardiovascular toxicity, hepatotoxicity, toxicity in other organs, and biomonitoring of prenatal METH exposure, as well as the possible emergence of sensitization associated with METH. We proposed the importance of gut microbiota in studying prenatal METH exposure. There is rising evidence of the adverse effects of METH exposure during pregnancy, which are of significant concern.

## Introduction

Illicit drug abuse has increasingly become a public health and social concern, worldwide. Methamphetamine (METH) is a serious international public health concern due to its high potential for addiction and the risk of long-lasting injury to multiple organs. Because of its anorexigenic and body-weight reducing effects, METH is in some cases used even during pregnancy to maintain body image. Additionally, its psychoactive and hallucinogenic effects greatly increase the risk of promiscuity and unwanted pregnancy. Mounting evidence indicates that prenatal METH exposure is rising (Marwick, 2000; Wouldes and Lester, 2019). In 1994, METH accounted for 8% of admitted pregnant women, which rose to 24% by 2006 (Terplan et al., 2009). Women comprise a sizeable and growing proportion of METH abusers, and they begin METH use at younger ages and seem more dependent on METH (Dluzen and Liu, 2008). These women are likely to continue drug abuse and become potential METH abusers during pregnancy.

Structurally, METH is identical to monoamine neurotransmitters and induces neurotoxicity, cardiovascular toxicity, and hepatotoxicity (Carvalho et al., 2012; Varga et al., 2015; Kim et al., 2020). METH easily crosses the placental barrier and even accumulates in breast milk (Little and Vanbeveren, 1996; Chomchai et al., 2016). The research conducted by David indicated that METH crossed the placenta within 30 s of its administration in pregnant sheep (Burchfield et al., 1991). In addition, it is reported that the concentration of METH was higher in breast milk than in maternal plasma (Steiner et al., 1984) and METH can be detected in breast milk following recreational use of METH (Bartu et al., 2009). Thus prenatal METH exposure may affect maternal health and also increase the risk of pregnancy complications like hypertension and preeclampsia during pregnancy as well as cause short- or long-term damage to the fetus. Silvia reported a case of neonatal brain malformation due to prenatal METH abuse in Philippines, and METH was detected in the urine of the mother and the newborn (Maya-Enero et al., 2018). Eugeni also reported a similar case, in which a full-term newborn born suffered seizures and severe neurological symptoms shortly after birth due to prenatal METH exposure, followed by severe liver insufficiency (Maranella et al., 2019). Understanding the impact of prenatal METH exposure on the fetus and the underlying mechanisms is crucial for early detection, intervention, and postpartum treatment of high-risk newborns.

This review summarizes the toxicological effects of METH exposure during pregnancy on the offspring. We describe neurotoxicity, cardiovascular toxicity, hepatotoxicity, and toxicity in other organs, as well as biomonitoring prenatal METH exposure and the possible emergence of METH-associated sensitization. We also highlight the value of gut microbiota in the study of prenatal METH exposure.

## Neurotoxicity

METH is neurotoxic, making prenatal exposure particularly concerning for fetal brain development. Neuronal impairment by METH affects microglia and causes oxidative stress, transcription factor activation, mitochondrial metabolism dysfunction, DNA damage, excitatory toxicity, apoptosis, and neuroinflammation (Xie et al., 2018; Xie et al., 2018; Xue et al., 2019; Kim et al., 2020; Tan et al., 2020). A relationship between prenatal METH exposure and neurotoxicity in offspring has been reported. A prospective cohort study in the United States and New Zealand found that METH exposure in pregnancy is associated with greater stress/abstinence, physiological stress, and central nervous system stress in the offspring (Lagasse et al., 2011). METH exposure increases emotional reactivity, anxiety, depression, attention disorders, withdrawal behavior, while externalization and attention-deficit/hyperactivity disorders are significant by 5 years of age (Lagasse et al., 2012). Additionally, prenatal METH exposure is associated with poorer quality of movement Smith et al. (2008), Lagasse et al. (2011), Wouldes et al. (2014), lower arousal and higher lethargy scores Smith et al. (2008), Paz et al. (2009), Kiblawi et al. (2014), poorer personal-social ability, motor coordination ability Dyk et al. (2014), poorer behavioral and executive function Himes et al. 2014, poorer academic achievements Cernerud et al. (1996), Behnke et al. (2013) and decreased self-regulation Lacy et al. (2014) in offspring. Infant cognition and development are inseparable from mother’s care and those who abuse METH during pregnancy show greater parenting stress and depressive symptoms (Liles et al., 2012). These mothers were more likely to have a psychiatric disorder/emotional illness and less prenatal care, and were less likely to breastfeed (Shah et al., 2012). All of these may affect the mother's parenting behavior and adversely affect offspring development, especially nervous system development.

Consistent with this, offspring of women exposed to METH during pregnancy exhibited neuroimaging differences. Chang found that children exposed to METH prenatally exhibit smaller subcortical volumes and associated neurocognitive deficits Chang et al. (2004), which was consistent with results from Sowell's study (Sowell et al., 2010). Studies have found structural alterations in brain areas due to METH exposure, including reduced striatal and hippocampal volume. Compared to unexposed children, children prenatally exposed to METH had lower apparent diffusion coefficient in the frontal and parietal white matter. They showed higher fractional anisotropy in the left frontal white matter, indicating less myelination and higher dendritic or spine density in the brain (Cloak et al., 2009; Colby et al., 2012). Analysis of brain development network pattern showed that relative to children without METH exposure, those prenatally exposed to METH had alterations in white matter microstructure and maturation, which closely correlated with functional abnormalities. Using rat models, Zoubkova showed that prenatal METH exposure alters the expression of thousands of genes in the striatum and hippocampus (Zoubková et al., 2019). Dong’s team found that maternal METH exposure altered the expression of genes involved in neurogenesis, axon guidance, neuron migration, and neural development circuit synapse in offspring (Dong et al., 2018). Peter G found that *in utero* METH exposure enhances oxidative DNA lesion 7,8-dihydro-8-oxoguanine (8-oxoG) in CD-1 fetal mouse brain, and causes long-term postnatal motor coordination deficits. Oxoguanine glycosylase 1 (ogg1) and Cockayne syndrome B (CSB) protein, which is involved in the repair of oxidatively damaged DNA, protect the fetus from xenobiotic-enhanced DNA oxidation and postnatal functional deficits Wong et al. (2008), Mccallum et al. (2011), suggesting that oxidatively damaged DNA may modulate neurotoxicity risk in offspring exposed to prenatal METH. METH regulates multiple neurotransmitters, including dopamine, glutamate, γ-aminobutyric acid (GABA), acetylcholine, and 5-hydroxytryptamine (5-HT). Similarly, offspring also showed the same trend. Siegel and his team found that METH exposure increased acetylcholine neuron density in regions of the basal forebrain and the area occupied by acetylcholine axons in the hippocampus in female offspring, but not the density of GABA cells or total neurons in the basal forebrain (Siegel et al., 2011). In contrast, another study found that METH-exposure decreased GABA levels but increased basal levels of monoamines and glutamate in caudate-putamen, infralimbic cortex, and ventral hippocampus (Fujáková-Lipski et al., 2017). Little is known about the effects on neurotransmitters or neurotransmitter systems in METH-exposed offspring. The current study revealed that prenatal METH exposure may disrupt the excitation/inhibition balance in the brain, which is associated with emotional and stress-related impairments, as well as learning and motor processes problems. Additionally, the cholinergic system has been associated with cognitive function. More studies are needed to confirm that the change of emotional reaction and behavior observed in population-cohorts is related to changes in brain transmitter levels.

## Cardiovascular Toxicity

The impact of METH on cardiovascular health is a rapidly developing research area. However, the cardiovascular effects of prenatal METH exposure in adult offspring have received limited attention. An animal study conducted by Rorabaugh's group found that prenatal METH exposure did not significantly affect infarct size, preischemic contractile function, or postischemic recovery of contractile function in male hearts. However, females exhibited significantly larger infarcts and significantly elevated end-diastolic pressure during recovery from ischemia. Further studies showed that METH significantly reduced protein kinase Cε expression and Akt phosphorylation in female hearts but had no effect on these cardioprotective proteins in male hearts (Rorabaugh et al., 2016). A recent study by Allison indicated that in adult male offspring, but not adult female offspring, endothelium-dependent relaxation to acetylcholine was impaired following METH-exposure, but there was no effect on systolic blood pressure in offspring of either gender (Harrison et al., 2021). Similar results have been found in METH-exposed adult rats (Rorabaugh et al., 2017). In adult individuals, METH-dependency has been associated with significantly reduced heart rate variability, reduced parasympathetic activity, diminished heartbeat complexity Henry et al. (2012), reduced stroke volume, and left ventricular end-diastolic volume, relative to healthy adults (Freeling and Mcfadden, 2020). Besides, other histopathological changes like cardiomyocyte hypertrophy and myocardial interstitial fibrosis were observed Sun et al. (2019), which might advance to cardiac ischemia, myocardial infarction, and cardiomyopathy Panenka et al. (2013), that may be life-threatening (Li et al., 2012; Zamanian et al., 2018; Lucena, 2019; Morentin et al., 2019). However, the mechanism remains undetermined. Cardiomyocyte apoptosis Chen et al. (2016), Sun et al. (2019) and neurotransmitter perturbation Hassan et al. (2015) contribute to METH-induced cardiovascular toxicity. Remarkably, cardiovascular toxicity due to prenatal METH exposure in adult offspring exhibits significant sex differences but the reason for this discrepancy is unknown. Shen reported that chronically abused METH disrupts the hypothalamic-pituitary-ovarian axis in women Shen et al. (2014), indicating that METH exposure during pregnancy may affect estrogen secretion in female offspring and damage its protective effects on the heart. Gender has different effects on the pharmacokinetics of METH in the placenta. Rambousek found that METH concentrations in the plasma and brain of female rats are significantly higher than in males, suggesting a greater risk of addiction and toxicity relative to male rats (Rambousek et al., 2014).

## Hepatotoxicity

METH causes multiple organ damage in abusers and frequently affects the liver. Due to METH’s low plasma protein binding, the liver is its main metabolic site (Kraemer and Maurer, 2002). In a population study, positron emission tomography scanning of 19 individuals for METH distribution Volkow et al. (2010) found that the liver has among the highest METH accumulation. Hepatocyte swelling and vacuolar-like changes were observed in livers of adults after METH abuse, with lysosomal swelling and elevated levels of transaminases (Pontes et al., 2008; Qu et al., 2020). However, few studies have examined hepatotoxic effects of prenatal METH. Liver biopsies revealed marked intralobular cholestasis with a significant acute and chronic portal and intralobular inflammation, including eosinophils (Dahshan, 2009). Another case found that prenatal METH exposure in neonates causes hepatic insufficiency, coagulopathy, and thrombocytopenia Maranella et al. (2019) and in both cases, newborns were 35 weeks premature, and one of the mothers had a clear 6-year history of substance abuse. It is difficult to determine if this is related to long-term exposure, exposure dose, or gestational age. More cases and related studies are needed to clarify the mode and mechanism of damage to offspring liver by prenatal METH exposure.

## Toxicity in Other Organs

METH exposure during pregnancy has been shown to cause toxicity in other organs. Solomiia’s study showed that episodic maternal exposure to METH causes lifelong impairment in glucose homeostasis in female, but not male offspring, as manifested by impaired insulin secretion by pancreatic B-cells. Sex-specific epigenetics of the serotonin related gene regulatory networks upstream of the transcription factor PET1/FEV may determine reduced reprogrammed insulin secretion (Korchynska et al., 2020). Prenatal METH exposure is reported to cause olfactory function deficits, probably associated with nuclear factor E2-related factor 2 (NRF2) (Ramkissoon and Wells, 2013). A study by Lorena G revealed that METH adversely affects growth pattern in postnatal rats and temporarily affects the dopaminergic system in the developing retina Rodrigues et al. (2006), suggesting that METH exposure during pregnancy may affect retinal development.

## Behavioral Sensitization and Cross-Sensitization

Continued drug abuse enhances the motor-stimulant response to these drugs. This phenomenon is termed behavioral sensitization and includes enhanced behavioral response and stereotypical behavior (Steketee and Kalivas, 2011). Behavioral sensitization is commonly assessed by drug-seeking behavior (detected by conditioned place preference studies and self-administration) and monitoring motor activity (detected by Open-Field Test and Laboras Test). Extensive studies show that similar to other psychoactive drugs, METH exposure in adult individuals can lead to behavioral sensitization but the specific mechanism is unknown. Increased dopamine levels have been associated with METH sensitization Fukakusa et al. (2008), Lan et al. (2009) decreased dopamine D2 receptor (D2R), dopamine D3 receptor (D3R), and dopamine transporter (DAT) activity (Huang et al., 2020; Huang et al., 2020). Previous research showed that METH sensitization specifically increased prefrontal 5-HT serotonergic neurons responsiveness and alleviated behavioral sensitization in mice by activating 5-HT receptors Ago et al. (2008), while GABA influences METH-induced behavioral sensitization by suppressing prefrontal cortex Wearne and Cornish (2019) regulation. Besides, some studies have shown that μ-opioid systems modulates behavioral sensitization. The μ-opioid receptors (MORs) are reported to modulate the function of the dopamine system and are inhibited in the striatum Hipolito et al. (2008) and the nucleus accumbens (NAc) Chefer et al. (2009) of METH-sensitized mice. Shen found that repeated METH administration initiated behavioral sensitization in wild-type mice, and these changes were attenuated in μ-opioid knockout mice, suggesting that MORs regulate behavioral sensitization of METH Shen et al. (2010), which is consistent with Kuo’s findings that the role of the u-opioid receptor is associated with its location in the brain. Using a stereotactic injection technique, AAV-MORs was injected into the NAc or the ventral tegmental area (VTA) of adult mice and MORs overexpression in NAc and VTA differentially affected METH sensitization, with MORs in VTA enhancing behavioral sensitization while MORs in the NAc region had an antagonistic effect (Kuo et al., 2016). Mounting evidence suggests that neuroplasticity changes are related to behavioral sensitization. Repeated exposure to psychoactive drugs may cause neural adaptation in the brain, which mediates sensitization behavior. AC 1/8 are critical to mechanisms that subserve drug-induced behavioral sensitization and mediate nigrostriatal pathway METH sensitivity. Specifically, AC 1 and AC 8 isoform deficiency, which uniquely couple activity-dependent increases in intracellular calcium to cAMP/PKA pathways, enhances dorsal striatal dopaminergic tone and disrupts METH-induced regulation of dopamine levels and activation of DARPP-32 mediating locomotor sensitization behavior (Bosse et al., 2015). Moriguchi found that Junctophilin 3 (JP3) and JP4 double-knockout (JP-DKO) mice exhibit aberrant synaptic plasticity in the corticostriatal circuits and irregular METH-induced behavioral sensitization. Elevated calcineurin (CaN) and aberrant calcium/calmodulin-dependent protein kinase II (CaMKII) activities in the striatum of JP-DKO mice likely accounts for lack of METH-induced behavioral sensitization (Moriguchi et al., 2015). Increased pERK and δ fosb levels in NAc and the caudate nucleus is also accompanied by METH-induced behavioral sensitization (Li et al., 2016; Wen et al., 2020). Overall, affecting neuroplasticity and the level of phosphorylated protein in the brain may underlie METH-induced behavioral sensitization.

Not surprisingly, consistent with adults, prenatal exposure to METH can also induce behavioral sensitization in offspring. Prenatal METH exposure makes adult rats more sensitive to acute injection of the same or related drugs, and displayed higher drug-seeking behavior than both controls (Alamberová et al., 2012). In line with this view, Bubenikova discovered that prenatal exposure to METH, resulted in more sensitivity to an acute dose of METH in adult offspring, with significant changes in the mesolimbic dopaminergic system (Bubenikova Valesova et al., 2009). Furthermore, both brain monoamine function and behavior alterations were found by Weissman’s team, manifested as a significant decrease in square crossing and rearing in an open field (Weissman and Caldecott-Hazard, 1993). However, no behavioral sensitization to METH was founded in the research of Sato and Fujiwara, regardless of the repeated prenatal exposure to METH (Sato and Fujiwara, 1986). This might be explained by the differences in maternal exposure time and dose, suggesting that there is a specific developmental stage of the brain that is essential for behavioral sensitization to METH. Notably, Schutov indicated that the sensitivity to METH during prenatal development and in adulthood exerted sex-specific effects (Schutová et al., 2013). Sirova indicated that the combination of prenatal and postnatal METH exposure increases the risk of dopaminergic deficits by altering the activity of surface-expressed DATs, and that male mice were more sensitive. Additionally, changes in the fluidity of striatal membranes may significantly reduce the activity of surface-expressed DATs in female mice (Sirova et al., 2016). It should be noted that degeneration of dopaminergic neurons and monoamine neurotransmitter deficiency was observed in METH-exposed offspring (Salisbury et al., 2009). Changes in 5-HT and Dopamine levels were also found in previous studies (Tonge, 1973; Cabrera et al., 1993; Heller et al., 2001; Won et al., 2002). The serotonin system and monoamine neurotransmitters seem to play an important role in METH-induced behavioral sensitization.

Cross-sensitization related to METH also deserves further attention. The abuse of one drug can lead to increased susceptibility to another, a phenomenon called cross-sensitization. Table 1 Stanwood and Levitt (2003), Suzuki et al. (2003), Ruda Kucerova et al. (2018), Clifford et al. (2009), Alamberová et al. (2012), Lewis et al. (2013), Chiang et al. (2014), Lacy et al. (2014), Wong et al. (2014), Lacy et al. (2016) summarizes the cross-sensitization related to METH and shows that exposure to METH during pregnancy may cause abuse of many psychoactive drugs, including cocaine and morphine. Environmental factors like early mother-infant separation may also increase susceptibility of offspring to METH abuse. Further studies are needed to elucidate the mechanisms underlying METH-induced behavioral sensitization. The roles played by various brain regions in behavioral sensitization need to be established. The task is arduous due to limitations in behavioral sensitization-related testing and difficulties in defining quantitative standards. Nevertheless, behavioral sensitization may explain the occurrence of familial drug abuse and mixed drug abuse.

**TABLE 1:** Sensitization associated with METH.

Conditions of cross sensitization/Resistance	Performance	References
Prenatal nicotine exposure increases sensitivity to METH	Motor behavior and conditioned hyperactivity was enhanced, and BDNF in the marginal cortex was changed	Lacy et al. (2016)
Intravenous METH injection increased the motivation to self-inject METH	Rats self-injected with more METH	Lacy et al. (2014)
The stresses of early mother-infant separation lead to vulnerability to METH intake	Metabolic syndrome was prolonged, METH intake was increased,and MeCP2 immunoreactivity in NAc region was decreased	Lewis et al. (2013)
Prenatal lead exposure enhances METH sensitization in rats	Behavioral sensitization occurs faster	Clifford et al. (2009)
Prenatal exposure to morphine, buprenorphine and methadone enhanced METH-induced behavioral sensitization	Motor activity and CPP activity were significantly increased, and the expression of dopamine D1R was lower in the NAc, and cAMP was dose-dependent	Chiang et al. (2014)
Prenatal METH exposure induces tolerance to cocaine	Shorter time in CPP testing of drug chambers	Alamberová et al. (2012)
Catecholamine-resistant cardiogenic shock occurred after prenatal METH exposure to the fetus	Dopamine, epinephrine, and norepinephrine dose increases were unresponsive and remained sluggish by day 4, and catecholamines responded well by day 9	Stanwood and Levitt, (2003)
Prenatal and neonatal exposure to bisphenol a enhances METH-induced sensitization	BPA treatment significantly enhanced METH-induced hyperactivity and sensitization, and the function of dopamine D1 receptor was upregulated, and the G-protein in the limbic forebrain was activated, and the level of dopamine D1 receptor gene was significantly increased	Suzuki et al. (2003)
Prenatal exposure to modafinil leads to an increased susceptibility to METH sensitization	The total distance of motion increased significantly in open-field test	Ruda Kucerova et al. (2018)
Prenatal methadone treatment increases METH sensitization	Prenatal methadone exposure not only promoted the development of METH-induced motor behavioral sensitization, but also restored behavioral sensitization in adolescent rats	Wong et al. (2014)

## Biomonitoring to Assess Prenatal Exposure to METH

Assessing prenatal exposure to METH is crucial for early recognition and treatment. Self-reported history and biomonitoring are the two basic methods to identify drug users. Unfortunately, self-reported history suffers from a problem with authenticity and accuracy (Maisto Sa, 1990; Behnke et al., 2013). A Prospective cross-sectional screening accuracy study conducted by Steven indicated that none of five screening instruments for substance use in pregnancy tested showed both high sensitivity and high specificity (Ondersma et al., 2019). It is needed to provide a destigmatized healthcare environment to encourage pregnant women to disclose their substance use to improve the reliability and validity of self-reported history (Berra et al., 2019). Biomonitoring is another efficient means for early recognition of prenatal exposure to METH. Different biological materials reflect exposures that occur over a specific time period, and each of these has special advantages and disadvantages, with regards to accuracy exposure window and cost/benefit ratio. This article mainly describes the biological materials related to the fetus. High-performance liquid chromatography (HPLC) or gas chromatography (GC) are the gold standards for METH detection, while ELISA is used for rapid detection. Neonatal hair is a sensitive biomarker for cumulative exposure to drugs during the last trimester of intrauterine life. Technological advances have revealed that hair samples can be used for quantitation and quantification of drug abuse (Ostrea et al., 2001; Bar-Oz et al., 2003; Vinner et al., 2003; Garcia-Bournissen et al., 2007). A major advantage of neonatal hair is its availability for as long as 4–5 months of postnatal life (Bar-Oz et al., 2003), and is effective in predicting neonatal withdrawal syndrome (Vinner et al., 2003). However, the sensitivity of hair detection is limited by the length and color of hair and has a high false positive rate (Vinner et al., 2003). Moreover, hair samples from newborns are often sparse and parents may resist hair cutting (Bar-Oz et al., 2003).

Compared to hair, meconium is safe and easily accessible. Meconium analysis allows the detection of maternal drug use during the final 20 weeks of gestation, allowing detection of fetal chronic drug exposure. Previous studies shown that meconium is highly sensitive in detecting neonatal drug exposure (Ostrea et al., 1989; Moriya et al., 1994; Moore et al., 1998). However, a prospective cohort study of 80 mothers and infants, indicated that METH discontinuous and/or sporadic consumption during pregnancy may have negligible transplacental passage and hence negative results in meconium analysis (Joya et al., 2016). Twenty-three newborns with one or two hair shafts positive to drugs of abuse did not present drugs in their meconium. Lack of timeliness is the major drawback of meconium testing. The acquisition time is limited, and the excretion of meconium might be delayed. Because the amniotic fluid is already formed in the first weeks of pregnancy, the presence of drugs in this fluid may indicate exposure during early fetal life. However, this test is rarely used due to risk to the fetus. Montgomery analyzed 498 umbilical cord tissues for 5 commonly abused drugs and compared the results to those from meconium tests Montgomery et al. (2008) and found that umbilical cord tissue tests are sufficient for clinical determination of fetal exposure to METH and other drugs of abuse. Moreover, umbilical cord tissue tests have speedier turnover relative to meconium testing since meconium passing may take days. The value of the placenta as biological material in assessing prenatal METH exposure has been explored (Myllynen et al., 2005; Montgomery et al., 2008; Al-Saleh et al., 2011; Płotka et al., 2013). The placenta is a protective barrier for the fetus through which drugs must pass through, as well as the site for nutrient and substance exchange. The placenta is a matrix that reflects the character of constant contact with the mother and fetus, and has been used to assess long-term exposure (Myllynen et al., 2005; Esteban and Castaño, 2009). Xenobiotic-metabolizing enzymes and transporters are closely related to drug exposure and have potential biomarker applications (Myllynen et al., 2005). Up to now, fetal drug exposure is mainly detected after birth. Antenatal detection of drug abuse is of great value. Using GC-MS, Joya quantitatively detected drug abuse in placental tissue at 12 weeks of pregnancy (Joya et al., 2010). This is the first report to highlight drug abuse in the first trimester and relied on biological materials obtained from women who had voluntarily terminated pregnancy at 12 weeks and the technique is limited by its invasiveness and therefore cannot be used in normal pregnancy screening.

## Prenatal METH Exposure and Gut Microbiota

Gut microbiota are closely linked to human health, including immunity and early development. That METH abuse may affect the abundance and composition of gut microbiota is supported by observations that the propionate-producing genus Phascolarctobacterium was decreased and the family Ruminococcaceae increased in the METH-induced conditioned place preference group (Ning et al., 2017). Further study by Yang showed that there was a significant difference in gut microbiota between group high CPP (the eight rats with the highest CPP scores) and low CPP group (the eight with the lowest scores), which was specifically manifested by the significantly increased of Akkermansia in group high CPP. In addition, there were already significant differences in gut microbiota before CPP training, which suggested that gut microbiota may be the regulatory factor of METH-induced behavioral abnormalities and differences in sensitivity to METH (Yang et al., 2020). Another study showed that METH exposure increased pathogenic bacteria abundances but reduced the abundance of probiotics (Chen et al., 2021). Angoa found that METH and its analogs caused significant time-dependent and structural-dependent changes in the composition of gut microbiota, with Firmicutes and Bacteriodetes having the most significant changes (Angoa-Pérez et al., 2020). Interestingly, Forouzan found that METH-induced changes in the gut microbiota gradually recovered seven days after drug withdrawal (Forouzan et al., 2021). This might be related to the different dosages of the two research. It remains to be determined if the changes in gut microbiota induced by METH have a time effect. A cohort study with 381 men found that the use of METH led to an abnormal increase in proinflammatory gut microbiota, including some bacteria that produce neuroactive substances and those associated with HIV (Cook et al., 2019). Another research conducted by Xu examined the composition and diversity of gut microbiota in 45 patients with substance use disorders, indicating that there seems to be a substance-related change in gut microbiota (Xu et al., 2017). Numerous studies have shown that maternal microbiota during pregnancy profoundly impacts offspring (Vuillermin et al., 2017; Calatayud et al., 2019). We speculate that prenatal METH exposure and the consequences to offspring may be associate with gut microbiota. Further research is worth being conducted.

The “sterile uterine cavity hypothesis” posits that the human uterine cavity is a sterile environment. However, recent studies have found that the uterine cavity is not absolutely sterile, and microorganisms have been detected in the meconium and amniotic fluid (Aagaard et al., 2014; de Goffau et al., 2019; Theis et al., 2019). Studies have shown that mother-to-infant transmission of strains mainly comes from horizontal transfer of intestinal flora (Ferretti et al., 2018). Maternal microbiota is crucial for neurodevelopment and offspring behavior. Cross-feeding and fecal microbiota transplantation are the common methods used in research. The offspring will be left with specific gut microbial characteristics from biological mother at birth, while a permanent microbiota shift could also be shaped by nursing mother (Daft et al., 2015; Treichel et al., 2019). Factors in breast milk like IgA (SIgA form) Rogier et al. (2014) and salivated milk oligosaccharides (MHOs) Charbonneau et al. (2016) may shape the gut microbiome. Robertson found that endogenous omega-3 unsaturated fatty acid (PUFA) production in Fat-1 transgenic mice is insufficient, and PUFA deficiency in the mother during pregnancy or lactation has lasting impact on the offspring’s intestinal microbiota (Robertson et al., 2018). Many small mammals engage in coprophagy (feces consumption), which helps stabilize gut microbiota and maintain necessary gut microbial diversity and function, altering cognitive performance (Bo et al., 2020). The role of gut microbiota was directly confirmed by maternal fecal microbiota transplantation experiments. Researchers from Mount Icahn Sinai School of Medicine found that the brain structure of sterile mouse embryos was different from that of female mouse embryos containing normal flora. These offspring had impaired responses to heat, sound, and pressure. After colonizing sterile mice with *Clostridium*, abnormal brain development and behavior in offspring were alleviated (Vuong et al., 2020). Jašarević transplanted prenatally stressed maternal vaginal microbiota into mice pups delivered by caesarean section and restored the pup’s phenotype (Jasarevic et al., 2018). Gut microbiota has been shown to play an important role in behavioral abnormalities related to the neurodevelopmental disorders of the offspring due to maternal immune stress (Kim et al., 2017). Metagenomic detection and gut microbiota reconstruction showed that *Lactobacillus* reuteri can correct oxytocin levels and induce synaptic potentiation (LTP) in the ventral tegmental area to regulate social deficits and gut microbiota disorders in the offspring of mothers fed with high-fat diet (Buffington et al., 2016).

Much less is known about changes in gut microbiota and the harmful effects to offspring due maternal METH abuse during pregnancy. Past studies shown that cross-fostering may affect offspring changes caused by prenatal METH exposure, including sensory, motor, and cognitive learning disabilities (Hrubá et al., 2008; Pometlová et al., 2009; Yamamotová et al., 2010). Studies by Itzhak found that responses to conditioned fear, spontaneous movement, and time in black compartment were affected by cross-fostering (Itzhak et al., 2015). The gut microbiota has a significant role in the harmful effects to offspring due maternal METH abuse during pregnancy, though further investigation is needed.

The biological mechanism by which prenatal METH exposure affects offspring development is undetermined. More relevant studies are necessary to prove whether gut microbiota is involved and to uncover the underlying mechanism of communication between mother and infant through gut microbiota. Metabolites or bile acids related to the gut microbiota may act as a bridge (Al and Eberl, 2020; van Best et al., 2020). Exploration of gut microbiota may explain the mechanisms of prenatal METH exposure and manipulation of the gut microbiota may be effective in preventing and treating maternal METH exposure to offspring.

## Conclusion

**FIGURE 1:**
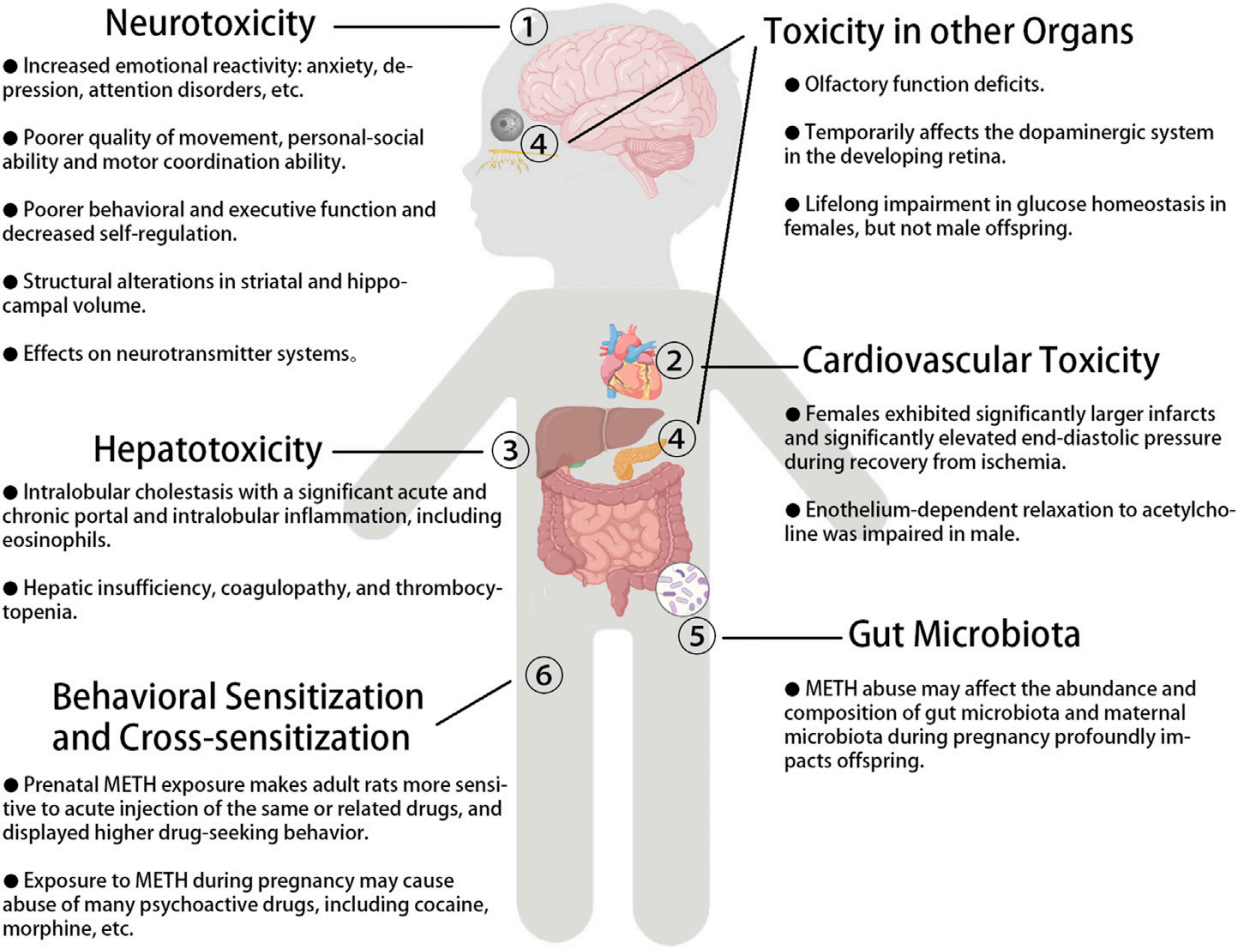
The adverse effects of prenatal METH exposure on the offspring.

There is growing evidence of a surge in METH use by women of childbearing age worldwide, with enormous socioeconomic harm. Current studies have associated prenatal METH exposure with neurotoxicity, cardiovascular toxicity, and hepatotoxicity in offspring and associated METH with behavioral-sensitization and cross-sensitization, which is shown in Figure 1. METH may affect fetal development *via* numerous mechanisms. Gut microbiota is a new research direction with great potential and value in underling mechanisms involved in intergenerational toxicity of prenatal METH exposure. Synergy between environmental susceptibility during pregnancy and METH exacerbates the risk. Continued monitoring of prenatal METH exposure is necessary. Long-term studies are needed to investigate the adverse effects and mechanisms underlying the effects of METH exposure during pregnancy as current studies are mainly on animal models. Increased efforts are needed to strengthen the publicity and education in pregnant women and provide psychosocial support to combat prenatal METH exposure and its effect on the newborn and the pregnant mother itself.
